# Community-Based Participatory Research: Its Role in Future Cancer Research and Public Health Practice

**DOI:** 10.5888/pcd10.120205

**Published:** 2013-05-16

**Authors:** Vanessa W. Simonds, Nina Wallerstein, Bonnie Duran, Malia Villegas

**Affiliations:** Author Affiliations: Vanessa W. Simonds, University of Iowa, Iowa City, Iowa; Bonnie Duran, University of Washington, Seattle, Washington; Malia Villegas, National Congress of American Indians Policy Research Center, Washington, DC.

## Abstract

The call for community-based participatory research approaches to address cancer health disparities is increasing as concern grows for the limited effectiveness of existing public health practice and research in communities that experience a disparate burden of disease. A national study of participatory research projects, Research for Improved Health, funded by the National Institutes of Health (2009–2013), identified 64 of 333 projects focused on cancer and demonstrated the potential impact participatory approaches can have in reducing cancer disparities. Several projects highlight the success of participatory approaches to cancer prevention and intervention in addressing many of the challenges of traditional practice and research. Best practices include adapting interventions within local contexts, alleviating mistrust, supporting integration of local cultural knowledge, and training investigators from communities that experience cancer disparities. The national study has implications for expanding our understanding of the impact of participatory approaches on alleviating health disparities and aims to enhance our understanding of the barriers and facilitators to effective community-based participatory research.

## Introduction

Cancer accounts for nearly 1 in 4 deaths in the United States (1). Although cancer mortality rates have decreased overall, these decreases are not as remarkable for racial/ethnic minority populations, who are diagnosed at later stages and have poorer survival rates compared with non-Hispanic whites (1). This disparity exists for cancers that can be detected early through screening, such as colorectal cancer, breast cancer, and cervical cancer. Disparities in mortality are likely related to lack of prevention and detection efforts (2).

Challenges to reducing cancer health disparities through public health practice and applied prevention research are vast and include multilevel intersecting factors. Widening socioeconomic and racial/ethnic health disparities documented in the past 20 years (3) — the chasm in the quality of health care availability, delivery, and accessibility (2), and the extended time it takes for research findings to translate into practice (4) — have created a national urgency to design effective interventions and applied prevention research, including an increased emphasis by the National Institutes of Health (NIH) on public health importance and impact.

Community-based participatory research (CBPR) has been identified as an approach in creating interventions and prevention strategies to reduce health disparities (5,6) and was highlighted as a core strategy in the first national NIH Summit to Eliminate Disparities (7). CBPR incorporates values and strategies to promote collaborative inquiry based on community-identified issues, equitable partnerships, and structures for participation; it also seeks to apply research to practice and policy for social change and reduced disparities (5). Within cancer and chronic disease prevention, CBPR provides strategies for contextualizing interventions to promote external validity across diverse communities and incorporates culturally centered indigenous knowledge with evidence-based sociobehavioral theory for comprehensive interventions (8). CBPR addresses mistrust between academia, public health agencies, and communities through reciprocal learning and the challenges of translating and sustaining interventions within specific community contexts to improve health (9). This article presents the extent of the use of CBPR within cancer prevention and control research, uses examples of CBPR cancer research to highlight emerging best practices, and showcases the potential for uncovering further best practices through a national cross-site CBPR study.

## CBPR Gaining Support and Recognition Within Cancer Research

The acknowledgment that traditional research methods have had limited effectiveness in alleviating health inequities has led the National Cancer Institute (NCI), other NIH institutes, the Centers for Disease Control and Prevention (CDC), the American Cancer Society, and large health foundations to cultivate comprehensive and participatory research approaches (5,10). Within NCI, the Center to Reduce Cancer Health Disparities is integral to NCI’s efforts to reduce the unequal burdens of cancer. The 2006 NCI strategic plan states the importance of community-based research for addressing health disparities and working with communities to address their needs (11). The Community Networks Program (CNP) evolved from several earlier initiatives. From 2005 through 2010, NCI funded 25 CNPs to support community-based participatory education, training, and research. A survey, conducted in 2010 (with responses by 22 of 25 CNPs) showed high operationalization of many CBPR principles, the most notable being building on community identity and strengths, co-learning, capacity development, and balance between research and practice (12). Resource and power sharing were more of a challenge, as was intervention sustainability, though the commitment to CBPR was reaffirmed. In 2010, NCI funded the current 23 CNP centers.

## Research for Improved Health: A National Study of CBPR Partnerships

With the increase in support for CBPR comes the need for understanding the characteristics of partners and partnerships in the CBPR process that best facilitate improved health outcomes. The Research for Improved Health (RIH) study is an NIH-funded study (2009–2013) designed to examine the variability of CBPR and community engagement projects nationwide, to assess facilitators and barriers to effective CBPR, and to identify correlations between select CBPR partnering processes and outcomes. As a partnership between the National Congress of American Indians Policy Research Center, the University of New Mexico, and the University of Washington, this cross-site study is a mixed-methods design integrating a web-based survey of CBPR projects nationwide and 7 in-depth case studies across diverse ethnic/minority populations and many disease outcomes (13).

In the first year of the RIH study, we documented the universe of federally funded CBPR projects in NIH’s 2009 RePORTER (Research Portfolio Online Reporting Tools) database. The inclusion criteria were studies that mentioned terms related to “CBPR,” “participatory,” “action,” or “engaged” research in the abstract and which had 2 or more years of funding still to be completed. In addition, 36 current projects funded by the Native American Research Centers for Health (NARCH) were added to the sample; these projects were not in the RePORTER database. (NCI had shown a previous commitment to NARCH of more than $1.2 million from 2002–2008.) However, the 38 projects undertaken by CDC’s Prevention Research Centers, 5 of which focused on cancer, were in the RePORTER database. We excluded smaller pilot grants, training grants, and R03 and R21 mechanisms (Table) because we wanted to focus on long-term projects that would allow us to collect data to answer our research questions about the added value of the partnership processes to promoting CBPR and health outcomes. The RIH study had a final sample of 333 CBPR projects, which then became the denominator for the Internet survey of partnerships that was administered during 2011–2012.

**Table:** Sample of Cancer-Related Community-Based Participatory Research Projects (n = 64), Research for Improved Health Study, National Institutes of Health (NIH), 2009^a^^,^ b

NIH Grant Type^c^	National Cancer Institute	National Institute on Minority Health and Health Disparities	Other National Institutes of Health	Centers for Disease Control and Prevention Prevention Research Centers	No. (%)
R01, n	13	1	2	NA	16 (25.0)
R24, n	0	9	1	NA	10 (15.6)
RC1/RC2, n	2 RC1	3 RC2	0	NA	5 (7.8)
U01/U54/U48, n	25	0	3	5 U48	33 (51.6)
Total, n (%)	40 (62.5)	13 (20.3)	6 (9.4)	5 (7.8)	64 (100)

Abbreviation: NA, not applicable.

a Does not include 5 projects that mention cancer as part of a list of chronic conditions potentially affected or the 9 REACH (Racial and Ethnic Approaches to Community Health) grants that focus on cancer or tobacco use cessation awarded by the Centers for Disease Control and Prevention.

b The full decision-making tree for selecting the community-based participatory research projects identified in the 2009 NIH RePORTER (Research Portfolio Online Reporting Tools) database is available from the investigators.

c R01: Research Project Grant Program is not limited in dollars, and grants are usually awarded for 3 to 5 years; these grants are used to support well-defined projects led by an experienced investigator. R24: Resource-Related Research Projects funds projects of $500,000 or more per year; these grants are used to provide resources for complex problems or to build research infrastructure. RC1: Challenge Grants in Health and Science Research were funded in 2009 for up to 2 years with budgets of up to $500,000 per year; these grants were used for high impact in biomedical or behavioral science. RC2: High Impact Research and Research Infrastructure Program funds projects with budgets greater than $500,000 per year for 2 years; these grants support high-impact ideas that may lay the foundation for new fields of investigation. U01: Research Project Cooperative Agreements are grants that have no specific dollar amount and are used to support well-defined projects led by an experienced investigator. U01/U54: Specialized Center Cooperative agreements are grants used to support any part of the full range of research and development from very basic to clinical using a multidisciplinary approach and usually focused on a specific disease. U48: Cooperative Agreements, Health Promotion and Disease Prevention Centers formed in cooperation with either a school of public health or medical school with a preventive medicine residency program. The centers are also funded through CDC and are committed to research that directly benefits communities. Additional NIH grant information is available from http://grants.nih.gov/grants/funding/funding_program.htm.

According to the abstracts, 64 projects were cancer-focused CBPR projects (Table). Of these, 40 were funded by NCI, 13 were funded by the National Institute on Minority Health and Health Disparities, 6 were funded by other NIH institutes, and 5 were funded by CDC’s Prevention Research Centers. Most of these studies focused on developing or implementing interventions; others identified cultural and other barriers to cancer prevention and screening. Topics included smoking cessation, screening, early detection, and vaccinations; use of different practice modalities and settings, such as lay health workers, patient navigators, workplace, clinic, school, and community settings; and targeted messaging and interactive technologies.

Finding such a large number of cancer research projects within the final sample of CBPR research projects raised the awareness of CBPR’s potential impact on reducing cancer health disparities. It also led to RIH decisions to include 2 cancer-focused case studies as part of the mixed-methods design. Data collection was completed at the end of 2012, and analyses are under way; accomplishments so far include the creation of the CBPR logic model (http://hsc.unm.edu/som/fcm/cpr/cbprmodel.shtml); publication of the research design (13) and existing instruments to measure CBPR constructs (13–15); and creation of 2 Internet survey instruments and qualitative interview and focus group guides (www.narch.ncaiprc.org and http://hsc.unm.edu/SOM/fcm/cpr/research.shtml), which include measures of CBPR principles (12), measures of trust, partnership synergy, and other metrics for evaluation of CBPR partnerships and their potential for affecting health outcomes.

## Examples of CBPR in Cancer Interventions

While the analyses of the national CBPR study is under way, with about one-fifth of the sample cancer-related, we hope to provide greater understanding of CBPR best practices within cancer prevention and control. The current state of cancer projects, however, does provide insight into emerging best practices, which we discuss below. These include adapting interventions within local contexts; developing trust; supporting integration of local cultural knowledge; and training investigators from communities that experience cancer disparities with combined goals of enhanced cultural legitimacy, community capacity, and sustainability of practices and programs.

### Adapting interventions within cultural and local contexts

Many CBPR cancer research projects have underscored the importance of identifying appropriate local contexts and understandings to ground their research, such as using salons and barber shops as effective sites for communicating health information to community members (6). From 2004 through 2009, Faith Moves Mountains, an R01-funded faith-based intervention, used the central role of the church and lay health advisors as an effective means for promoting cancer prevention (16). Compared with the control group, intervention participants had more than twice the likelihood of reporting receipt of a Papanicolaou test postintervention. This project also created the infrastructure for subsequent cancer prevention grants. From 2002 through 2008, the Colorectal Cancer Screening Intervention Trial used community health workers among African American community members first in Atlanta and later throughout Georgia. In 2012, this partnership joined forces with the National Black Leadership Initiative on Cancer to disseminate the intervention to 20 locations throughout the nation (17). Community health workers in their project have adapted the intervention to their communities, which ensures that the social context is addressed and enhances the likelihood of sustainability (6).

### Taking time to build trust — funding mechanisms that support authentic CBPR

In communities that have had negative experiences with universities, mistrust is a barrier to research participation, and it constrains innovation in eliminating health disparities. CBPR recognizes the importance of reducing levels of mistrust (6) and the time it takes to develop trust. For example, in 2005, the University of New Mexico’s Cancer Center launched its culturally appropriate leadership institutes and Cancer 101 workshops to connect Native American and Hispanic communities to cancer prevention and treatment resources and to share strengths across communities (http://cancer.unm.edu/reaching-out). These long-term educational efforts have proved successful in providing a foundation to initiate research projects, such as exploring cultural knowledge and beliefs about cancer; assessing facilitators and barriers to cancer prevention, screening, and treatment; and more recently, launching screening research interventions.

Many CBPR studies have cited the importance of pilot or planning grants for assisting in developing community trust and sustainability (18). The Community Grants Program developed in 2007 through the Carolina Community Network by NCI awarded small grants to allow community-academic partnerships time to build trust, establish objectives, and develop projects that address cancer health disparities, with a goal of sustainability after initial funding (19).

### Supporting integration of local cultural knowledge

CBPR practitioners are likely to appreciate community assets and work with communities to codevelop research that merges evidence-based practices with local wisdom (20). Several CBPR cancer prevention interventions have used trusted community members to provide cancer resources and information through their social networks in a manner that not only follows cultural protocols but also incorporates cultural wisdom so that community members will be most receptive to the health promotion messages. This codevelopment also creates opportunity for sustainability and community ownership (6). For example, these culturally centered community health worker models have shown favorable results, including effectively communicating information about prevention of prostate cancer (6) and cervical cancer (18). Results from the lay health worker model used in a Vietnamese community showed a significant increase in the receipt of a Papanicolaou test (65.8% to 81.8%) in the intervention group from preintervention to postintervention; the increase was also significantly greater in the intervention group than the control group, which did not use community health workers (18).

Community members coanalyzing data with professionals can provide insight into data interpretation; coanalysis can also build community capacity and collaboratively identify viable solutions to health issues (21). One of the RIH case studies is an R01 project in Chinatown, San Francisco, which has been translating effective cultural strategies within Vietnamese communities for using lay health workers to enhance cancer screening (18) among the Chinese immigrant and Chinese American population.

### Training investigators from communities experiencing cancer inequities

Another way to contextualize research and increase community capacity is to train people from communities experiencing cancer health inequities to become researchers. NIH centers prioritize training, and many CBPR research projects have provided opportunities for students to address health disparities in their own communities. A recent survey of junior investigators engaged in CBPR through the NCI’s CNP centers showed promising results on research productivity, engagement, and satisfaction with CBPR from investigators from underrepresented backgrounds (22). Researchers who possess in-depth knowledge, understanding, and relationships in the communities with whom they are working can bridge the academic and community contexts. Through support from the ‘Imi Hale — Native Hawaiian Cancer Network, more than 14 Native Hawaiians had completed or enrolled in doctoral training, and 30 completed or enrolled in medical school or master’s degree programs as of 2008 (23).

## The Future of CBPR in Cancer Research and Public Health Practice

We have illustrated the emphasis that NIH and NCI have placed on using CBPR approaches to address cancer-related health concerns. This emphasis is also exemplified by recent calls for funding; since fall 2011, the American Cancer Society has issued a Request for Application twice each year for CBPR pilot studies to build partnerships and gather preliminary data for future NIH applications. Collaborative research awards, including partnership development, also have been created at the state level, such as the California Breast Cancer Research Program and the University of California Tobacco-Related Disease Research Program. 

Despite the growing interest in CBPR and its promise for enhancing the effectiveness of interventions, there remains the challenge to better understand the characteristics of partnerships and participation by community members, public health professionals, and researchers to most effectively produce health outcomes. The literature of CPBR has documented systemic outcomes such as policy changes, practice and program changes for greater sustainability and equity, and community capacity and empowerment outcomes, all of which contribute to health outcomes (5,9,24). NIH has also touted the benefits of community participation in research design, enhanced recruitment and retention into studies, development of culturally appropriate measures, and assurance of cultural centeredness and intervention feasibility.

The understanding of how CBPR processes impact health disparities still needs to be strengthened. Learning how the added value of participatory processes works can improve our capacity to reduce cancer and other chronic disease disparities. To that end, the RIH national CBPR analyses will enhance our understanding about the scientific contribution of CBPR. Incorporating trust as a core CBPR dimension, for example, the national study has developed a new trust scale, which is currently being coanalyzed with the in-depth exploration of trust in the case studies. In addition, separate analyses of CBPR projects addressing cancer-related or other chronic disease disparities nationwide will be able to look at how community involvement enables communities to strengthen their own support structures for people living with cancer or how academic–community partnerships that have deep roots in the community might better address peoples’ fears of cancer screening and diagnosis.

To promote increased effectiveness of CBPR within cancer prevention and control, with applicability also for other chronic disease prevention, we have named several emerging best practices, with a hope for others to be incorporated in the future:

1. Identify local settings and community beliefs and knowledge to promote adaptation of interventions within local contexts.
2. Continue to recognize the importance of building relationships and trust with unique diverse communities and agency and practice organizations. This recognition may entail strengthening linked grant awards that can support partnership development, education, and research.
3. Support interventions that incorporate cultural, local, and indigenous knowledge with sociobehavioral theory and evidence from the literature.
4. Train community members as researchers and agents of change.

In addition, we recommend using the CBPR conceptual model, using the metrics and instruments from the RIH national study to evaluate partnerships, assessing both CBPR processes as well as intermediate outcomes, such as systems, policy, and practice changes that can lead to improved health status. The potential for systematic collective reflection based on constructs within the model itself may be a best practice for partnerships to assess their history, their current status of partnering (ie, their level of power sharing or collective decision making), and their own best practices for reaching their vision of their partnerships now or in the future (25). With these recommendations, the upward trend to engage community members, public health professionals, and researchers can support the reciprocal learning needed to alleviate cancer and chronic disease health disparities over time.

## References

[1] Siegel R , Naishadham D , Jemal A . Cancer statistics, 2012. CA Cancer J Clin 2012;62(1):10–29. 10.3322/caac.20138 22237781

[2] Paskett ED . Cancer health [corrected] disparities: moving from why they occur to how they can be prevented. J Clin Oncol 2012;30(4):354–6. Erratum in: J Clin Oncol 2012;30(10):1149. 10.1200/JCO.2011.39.5947 22184382

[3] US Department of Health and Human Services. National healthcare quality and disparities report 2011. Rockville (MD): Agency for Healthcare Research and Quality; 2012. http://www.ahrq.gov/research/findings/nhqrdr/nhqrdr11/qrdr11.html. Accessed November 14, 2012.

[4] Institute of Medicine. Crossing the quality chasm: a new health system for the 21st century. Washington (DC): National Academy Press; 2001. p. 337. 25057539

[5] Minkler M , Wallerstein N , editors. Community-based participatory research for health. 2nd edition. San Francisco (CA): Jossey-Bass; 2008. p. 508.

[6] Meade CD , Menard JM , Luque JS , Martinez-Tyson D , Gwede CK . Creating community-academic partnerships for cancer disparities research and health promotion. Health Promot Pract 2011;12(3):456–62. 10.1177/1524839909341035 19822724 PMC3653573

[7] Dankwa-Mullan I , Rhee KB , Williams K , Sanchez I , Sy FS , Stinson N Jr , The science of eliminating health disparities: summary and analysis of the NIH summit recommendations. Am J Public Health 2010;100(Suppl 1):S12–8. 10.2105/AJPH.2010.191619 20147660 PMC2837420

[8] Duran B , Wallerstein N , Miller WR . New approaches to alcohol interventions among American Indian and Latino communities: the experience of the Southwest Addictions Research Group. Alcohol Treat Q 2007;25(4):1–10. 10.1300/J020v25n04_01

[9] Wallerstein N , Duran B . Community-based participatory research contributions to intervention research: the intersection of science and practice to improve health equity. Am J Public Health 2010;100(Suppl 1):S40–6. 10.2105/AJPH.2009.184036 20147663 PMC2837458

[10] Green LW . Making research relevant: if it is an evidence-based practice, where’s the practice-based evidence? Fam Pract 2008;25(Suppl 1):i20–4. 10.1093/fampra/cmn055 18794201

[11] US Department of Health and Human Services. The NCI strategic plan for leading the nation to eliminate the suffering and death due to cancer. Washington (DC): National Cancer Institute; 2006. http://strategicplan.nci.nih.gov/pdf/nci_2007_strategic_plan.pdf. Accessed May 8, 2012.

[12] Braun KL , Nguyen TT , Tanjasiri SP , Campbell J , Heiney SP , Brandt HM , Operationalization of community-based participatory research principles: assessment of the National Cancer Institute’s Community Network Programs. Am J Public Health 2012;102(6):1195–203. 10.2105/AJPH.2011.300304 22095340 PMC3292685

[13] Hicks S , Duran B , Wallerstein N , Avila M , Belone L , Lucero J , Evaluating community-based participatory research to improve community-partnered science and community health. Prog Community Health Partnersh 2012;6(3):289–99. 10.1353/cpr.2012.0049 22982842 PMC3586244

[14] Sandoval JA , Lucero J , Oetzel J , Avila M , Belone L , Mau M , Process and outcome constructs for evaluating community-based participatory research projects: a matrix of existing measures. Health Educ Res 2012;27(4):680–90. 10.1093/her/cyr087 21940460 PMC3396879

[15] Pearson C , Duran B , Lucero J , Sandoval J , Oetzel J , Tafoya G , CBPR variable matrix: research for improved health in academic/community partnerships; 2011. http://www.ces4health.info/find-products/view-product.aspx?code=FWYC2L2T. Accessed July 25, 2012.

[16] Studts CR , Tarasenko YN , Schoenberg NE , Shelton BJ , Hatcher-Keller J , Dignan MB . A community-based randomized trial of a faith-placed intervention to reduce cervical cancer burden in Appalachia. Prev Med 2012;54(6):408–14. 10.1016/j.ypmed.2012.03.019 22498022 PMC3368037

[17] Smith SA , Blumenthal DS . Community health workers support community-based participatory research ethics: lessons learned along the research-to-practice-to-community continuum. J Health Care Poor Underserved 2012;23(4):77–87. 10.1353/hpu.2012.0156 23124502 PMC3586526

[18] Nguyen TT , McPhee SJ , Bui-Tong N , Luong TN , Ha-Iaconis T , Nguyen T , Community-based participatory research increases cervical cancer screening among Vietnamese-Americans. J Health Care Poor Underserved 2006;17(2 Suppl):31–54. 10.1353/hpu.2006.0078 16809874

[19] Vines AI , Teal R , Meyer C , Manning M , Godley P . Connecting community with campus to address cancer health disparities: a community grants program model. Prog Community Health Partnersh 2011;5(2):207–12. 10.1353/cpr.2011.0027 21623024 PMC3612535

[20] Wynn TA , Anderson-Lewis C , Johnson R , Hardy C , Hardin G , Walker S , Developing a community action plan to eliminate cancer disparities: lessons learned. Prog Community Health Partnersh 2011;5(2):161–8. 10.1353/cpr.2011.0013 21623018 PMC3600640

[21] Cashman SB , Adeky S , Allen AJ 3d , Corburn J , Israel BA , Montano J , The power and the promise: working with communities to analyze data, interpret findings, and get to outcomes. Am J Public Health 2008;98(8):1407–17. 10.2105/AJPH.2007.113571 18556617 PMC2446454

[22] Felder TM , Brandt HM , Armstead CA , Cavicchia PP , Braun KL , Adams SA , Creating a cadre of junior investigators to address the challenges of cancer-related health disparities: lessons learned from the Community Networks Program. J Cancer Educ 2012;27(3):409–17. 10.1007/s13187-012-0361-0 22528636 PMC3407323

[23] Braun KL , Tsark JU . Community-based research should be based in the community. Prog Community Health Partnersh 2008;2(4):271–3. 10.1353/cpr.0.0043 20208305 PMC2914233

[24] Minkler M , Garcia AP , Rubin V , Wallerstein N . Community-based participatory research: a strategy for building healthy communities and promoting health through policy change. Berkeley (CA): PolicyLink; 2012. http://www.policylink.org/atf/cf/%7B97C6D565-BB43-406D-A6D5-ECA3BBF35AF0%7D/CBPR.pdf. Accessed July 25, 2012.

[25] Trickett EJ , Beehler S , Deutsch C , Green LW , Hawe P , McLeroy K , Advancing the science of community-level interventions. Am J Public Health 2011;101(8):1410–9. 10.2105/AJPH.2010.300113 21680923 PMC3134512

